# Naringin as a non-antibiotic agent for multi-species oral biofilm control: *in vitro* antimicrobial mechanisms and *in vivo* safety in a rat caries model

**DOI:** 10.3389/fmicb.2025.1722083

**Published:** 2025-12-10

**Authors:** Jie Song, Mingqi Liu, Yizinigaer Yasen, Ying Zhao, Zeyu Wu, Jin Zhao

**Affiliations:** 1 Department of Cariology and Endodontics, The First Affiliated Hospital of Xinjiang Medical University (The Affiliated Stomatology Hospital of Xinjiang Medical University), Ürümqi, China; 2 Stomatology Disease Institute of Xinjiang Uyghur Autonomous Region, Ürümqi, China

**Keywords:** Naringin, dental caries, multi-species oral biofilm, non-antibiotic, oral dysbiosis, extracellular polysaccharides (EPS)

## Abstract

**Introduction:**

Dental caries is driven by dysbiosis of oral biofilms. Conventional antibiotics easily disrupt oral commensal balance, creating an urgent need for natural, non-antibiotic agents that can target cariogenic biofilms without causing ecological collapse.

**Methods:**

The antimicrobial and antibiofilm efficacy of Naringin was evaluated *in vitro* against planktonic and biofilm states of *Streptococcus mutans, Streptococcus sobrinus, Streptococcus sanguinis* (mono-species), and their multi-species consortium. Minimum inhibitory/bactericidal concentrations (MIC/MBC) and minimum biofilm inhibitory/reduction concentrations (MBIC/MBRC) were determined. Effects on acid production, extracellular polysaccharide (EPS) synthesis, and bacterial adhesion to hydroxyapatite (HAP) were mechanistically investigated. *In vivo*, a rat caries model induced by the multi-species consortium was topically treated with Naringin (2 × MIC, MIC, 1/2 × MIC) for 4 weeks. Caries lesions were evaluated using Keyes scoring and micro-computed tomography. Oral microbiota, serum biochemistry, and histopathology were analyzed for safety assessment.

**Results:**

Naringin exhibited potent, concentration-dependent antimicrobial activity. MICs were 1.00 mg/mL for *S. mutans* and *S. sanguinis*, 0.50 mg/mL for *S. sobrinus*, and 0.50 mg/mL for the multi-species consortium. Naringin at MBIC (2 mg/mL for multi-species) significantly disrupted biofilm architecture, reduced viable bacteria, and inhibited EPS synthesis. It maintained biofilm pH above 5.5 (the critical threshold for enamel demineralization), inhibited lactate production, and reduced multi-species bacterial adhesion to HAP by 68.3% at MIC. *In vivo*, Naringin (MIC) significantly reduced Keyes scores on smooth and sulcal surfaces by over 60%, preserved enamel integrity, and rebalanced the oral microbiota without inducing mucosal irritation or systemic toxicity.

**Discussion:**

Naringin, a natural non-antibiotic agent, effectively inhibits the “adhesion-biofilm-acid-EPS” cascade of multi-species cariogenic biofilms. Its selective efficacy against pathogens and favorable *in vivo* safety profile position it as a promising ecological agent for caries prevention by addressing oral dysbiosis at its root.

## Graphical Abstract

**Figure:**
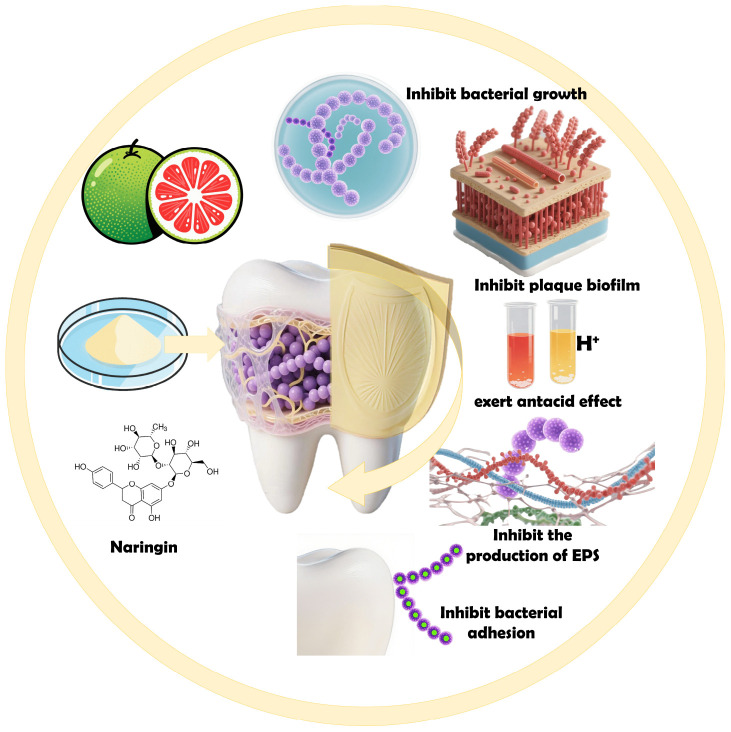
Mechanism of action and therapeutic applications of Naringin in the prevention and treatment of dental caries.

## 1 Introduction

Despite significant advancements in understanding the pathogenesis, early prediction, population-level prevention, and clinical management of dental caries-fueled by modern scientific and technological advances-dental caries remains a persistent challenge as one of the most prevalent and burdensome oral diseases globally. Indeed, data from The Lancet’s Global Burden of Disease Study (GBD) 2021 underscores that dental caries imposes a severe epidemic burden worldwide. From 1990 to 2021, the global burden of dental caries exhibited a distinct “differentiated improvement” trend: the age-standardized rate (ASR) and disability-adjusted life-year (DALY) rate of untreated permanent tooth caries declined, providing evidence for the efficacy of specific preventive and therapeutic interventions. Notably, the prevalence and DALY rate of untreated deciduous tooth caries remained stable over this 31-years period, without any substantial improvement. A considerable proportion of the global population remains at risk of progression of dental caries to complex lesions, largely due to delays in timely clinical intervention (Cheng et al., 2022; Bernabe et al., 2025; Wu et al., 2025). Streptococci are the most commonly isolated bacteria in dental caries, characterized by strong acid-producing and acid-tolerant capabilities. Moreover, they can synthesize water-insoluble glucans, which facilitate their adhesion to tooth surfaces, co-aggregation with other bacteria, and ultimately the formation of pathogenic biofilms (Van Houte, 1994). Specifically, dental caries arises from dysbiosis in oral biofilms, key oral streptococci such as *Streptococcus mutans*, *Streptococcus sobrinus*, and the early colonizer *Streptococcus sanguinis* dominate through coordinated virulence mechanisms, including extracellular polysaccharides (EPS) synthesis, acidogenesis, and adhesive colonization (Ye et al., 2025; Hajishengallis et al., 2023).

With the development of dentistry toward early diagnosis and prevention of dental caries, it is particularly important to target the etiological factors with active and effective measures to control dental plaque, reverse the imbalance between enamel demineralization and remineralization, and prevent the progression and spread of dental caries to the deep parts of the teeth. Pharmacological and remineralization therapies can arrest or eliminate early carious lesions, which are highly beneficial for caries prevention and control across the entire population and lifespan-particularly in oral hygiene management for special populations. In pharmacological prevention strategies, antibiotics have exerted profound impacts on caries control but are constrained by significant limitations. Fluoride-based interventions face challenges such as the emergence of fluoride-resistant strains, dose-dependent toxicity, and limited remineralization capacity in advanced lesions (Moghadam et al., 2020; Karygianni et al., 2016; Xu et al., 2024). Additionally, antimicrobial agents disrupt oral microbiota homeostasis and promote drug resistance, thereby limiting their long-term efficacy (Wang et al., 2017; Zhu et al., 2023). Natural antimicrobial agents represent an effective strategy to address antibiotic resistance. Consequently, these limitations have spurred research into natural polyphenolic compounds as alternative therapeutic candidates, particularly those with multi-target activity against biofilm architecture, acid production, and bacterial adhesion (Cao et al., 2024; Ren et al., 2025). Notably, natural medicines have emerged as a research hotspot in the prevention and treatment of early dental caries, attributed to their excellent antibacterial properties and ability to maintain the enamel demineralization-remineralization balance. With a wide variety of natural medicines, their active components and efficacy are complex and diverse, enabling them to perfectly tackle the issue of antibiotic resistance (Narwal et al., 2025; Pusporini et al., 2025).

Naringin, a dihydroflavonoid compound with the molecular formula C_27_H_32_O_14_, is a major flavanone glycoside predominantly extracted from citrus peels such as Pericarpium Citri Reticulatae Viride (Joshi et al., 2018). It is widely used as a flavoring agent, flavor enhancer, and food additive, and numerous studies have demonstrated its diverse biological activities, including anti-cancer, antibacterial, enzyme-inhibiting, and antioxidant properties. As an abundant dihydroflavonoid glycoside in citrus peels, Naringin has emerged as a promising non-antibiotic agent due to its potent antibacterial and antibiofilm activities (Tocmo et al., 2020; Posadino et al., 2024; Wei et al., 2025). Notably, it serves as a key active component in traditional Chinese medicinal herbs like Exocarpium Citri Grandis and Fructus Aurantii; the “Pharmacopeia of the China” (2025 edition) records that these herbs exert effects such as “dispersing cold, drying dampness, regulating qi, and resolving phlegm” or “regulating qi to broaden the middle energizer and relieving stagnation to reduce distension,” which may involve antibacterial actions by aiding in alleviating respiratory infections. Preliminary studies suggest that Naringin exerts its antibacterial effects through mechanisms such as disrupting bacterial cell membranes, inhibiting glucosyltransferase (GTF) activity, and modulating quorum sensing pathways, although its specific mechanism of action against multi-species cariogenic biofilms remains incompletely characterized (National Pharmacopoeia Commission, 2025; Han and Lee, 2022; Truchado et al., 2012). Additionally, its low cytotoxicity and high biocompatibility align with the growing demand for eco-friendly, microbiota-sparing oral care agents (Wei et al., 2025).

This study systematically evaluates Naringin’s antimicrobial mechanisms against planktonic and biofilm states of key cariogenic species, coupled with its *in vivo* safety profile in a rat caries model. By integrating *in vitro* analyses of bacterial growth kinetics, biofilm formation, acid production, and EPS architecture with *in vivo* assessments of caries progression and microbiota homeostasis, we aim to establish Naringin as a novel therapeutic agent for controlling multi-species oral biofilms while addressing the limitations of conventional antimicrobial strategies.

## 2 Materials and methods

### 2.1 Chemicals, bacterial strains, and experimental animals

Naringin was obtained from Shanghai Yuanye Biotechnology Co., Ltd., Brain Heart Infusion (BHI) medium and Mitis Salivarius Agar were purchased from BD and Beijing Land Bridge Technology Co., Ltd., respectively. Anaerobic generators and indicators were from Mitsubishi. Agar, hemin, vitamin K1 injection, and phosphate-buffered saline (PBS) were also used.

SPF-SD rats were procured from the Animal Experiment Center of Xinjiang Medical University, with experiments approved by the Institutional Animal Care and Use Committee (IACUC-JT-20240415-16). *Streptococcus mutans* UA159 (*S. mutans*), *Streptococcus sanguinis* (*S. sanguinis*), and *Streptococcus sobrinus* (*S. sobrinus*) were obtained from Institute of Microbiology, Guangdong Academy of Sciences, cultured in BHI broth at 37 °C for 24 h, and adjusted to 10^7^ CFU/mL before storage at −80 °C.

For medium preparation: 0.5% Hemin-vitamin K1 solution was prepared by dissolving 50 mg hemin in 100 mL double-distilled water, autoclaving, cooling to 50 °C, and mixing with 1 mL vitamin K1 injection. BHI liquid medium: 3.7 g BHI powder in 100 mL water was autoclaved, cooled, and supplemented with 0.1 mL hemin-vitamin K1 solution. BHI solid medium: 3.7 g BHI powder and 1.8 g agar in 100 mL water were autoclaved and mixed with 0.1 mL hemin-vitamin K1 solution. MSA solid medium: 9.0 g MSA basal medium in 100 mL water was autoclaved.

### 2.2 Antimicrobial activity against planktonic bacteria

#### 2.2.1 Mono-species MIC/MBC determination

Bacterial suspensions of *S. mutans*, *S. sanguinis*, *S. sobrinus* (OD_630_ = 0.2) were prepared following broth dilution protocol (Clinical and Laboratory Standards Institute [CLSI], 2023). Naringin was dissolved in DMSO and formulated with BHI broth containing 1% sucrose to a stock concentration of 16 mg/mL, then serially diluted to 8–0.125 mg/mL. Experimental groups included: Naringin (8–0.125 mg/mL); 0.12% CHX (positive control group); 0.1% DMSO (vehicle control group); BHI broth (negative control group). Each solution (100 μL) was mixed with 100 μL bacterial suspension in 96-well plates and incubated at 37 °C for 24 h. Absorbance at 595 nm was measured. The minimum inhibitory concentration (MIC) was defined as the concentration causing 50% growth inhibition. For MBC, 10 μL from wells with concentrations ≥MIC were plated on BHI agar. MBC was the lowest concentration with no visible colonies after 24 h incubation.

#### 2.2.2 Multi-species consortium MIC and MBC analysis

A multi-species consortium of *S. mutans*, *S. sanguinis*, and *S. sobrinus* (1:1:1 ratio) was prepared (Zhang et al., 2025). The consortium was cultured in BHI broth at 37 °C for 24 h. MIC and MBC determinations followed the same methods as in Section “2.2.1 Mono-species MIC/MBC determination.”

#### 2.2.3 Time-kill curve assay

To evaluate the dynamic antimicrobial efficacy of Naringin over time, time-kill curve assays were performed for *S. mutans*, *S. sanguinis*, and *S. sobrinus* as mono-species cultures and their multi-species consortium. Bacterial suspensions (mono-species or multi-species consortium) were mixed with Naringin solutions (8–0.125 mg/mL) in 96-well plates. Plates were incubated at 37 °C under microaerophilic conditions. Absorbance at 595 nm was measured at time points 1, 2, 3, 4, 6, 8, 10, 12, 16, 20, and 24 h. Viable counts (CFU/mL) were determined by plating serial dilutions.

### 2.3 Mono-species biofilm models for individual pathogen control

#### 2.3.1 Single-species biofilm formation and Naringin treatment

Following Chamlagain et al. (2024), MBIC was defined as the lowest Naringin concentration inhibiting ≥50% biofilm formation. MBRC was defined as the lowest concentration reducing ≥50% of preformed biofilms. Biofilms of *S. mutans*, *S. sanguinis*, and *S. sobrinus* were established in 96-well plates with 1% sucrose-BHI medium. For MBIC, Naringin (32–0.125 mg/mL) was added during inoculation. For MBRC, Naringin was applied to 24 h preformed biofilms. After 24 h incubation, biofilms were fixed, stained with crystal violet, and solubilized for A595 measurement.

#### 2.3.2 Biomass and viability analysis in mono-species biofilms

Biofilms were formed on coverslips and treated with Naringin at MBIC, 1/2MBIC, and 1/4MBIC concentrations. After treatment, biofilms were stained with LIVE/DEAD BacLight™ Kit and imaged by fluorescence microscopy. Three random fields per sample were imaged, and the ratio of live bacteria (SYTO 9^+^/PI^–^) to total bacteria (SYTO 9^+^) was analyzed using ImageJ software. Experiments were repeated three times with triplicate samples to compare inter-species responses to Naringin.

### 2.4 Multi-species biofilm control models for clinical relevance

#### 2.4.1 Establishment of tri-species caries biofilm model

*Streptococcus mutans*, *S. sanguinis*, and *S. sobrinus* were cocultured at 1:1:1 ratio on disks in BHI with 1% sucrose. Plates were incubated at 37 °C for 24 h. Biofilm maturity was verified by CLSM and crystal violet staining (Li Q. et al., 2023).

#### 2.4.2 Naringin-mediated disruption of multi-species biofilms

Mono- and multi-species biofilms were treated with Naringin (32–0.125 mg/mL) for 24 h. MBIC and MBRC were determined as in Section “2.3.1 Single-species biofilm formation and Naringin treatment.” Bacterial viability was assessed by LIVE/DEAD staining and CLSM.

### 2.5 Mechanistic comparison between mono-and multi-species biofilms

#### 2.5.1 Ultrastructural differences assessed by SEM

To decipher the differential effects of Naringin on mono-vs. multi-species biofilms, scanning electron microscopy (SEM) was employed to visualize ultrastructural changes in biofilm architecture and extracellular matrix composition. Biofilms were grown on saliva-coated coverslips, treated with Naringin (MBIC, 1/2MBIC, 1/4MBIC) for 24 h, then processed for SEM imaging.

#### 2.5.2 Fluorescent staining for glycocalyx analysis

To dissect the differential effects of Naringin on glycocalyx production in mono-vs. multi-species biofilms, fluorescent labeling was employed to visualize bacterial-EPS interactions. Biofilms were cultured with Alexa Fluor 647-dextran conjugate (Invitrogen) to label extracellular polysaccharides (EPS) (Guo et al., 2025) and stained with SYTO9 for bacteria. Samples were imaged by CLSM, and EPS-bacteria ratios were quantified.

#### 2.5.3 Effect of Naringin on the synthesis of EPS by cariogenic bacteria

Dextran standard curve was prepared using sulfuric acid-anthrone method. Planktonic bacteria were cultured with Naringin (MIC-1/8MIC) for 24 h. Insoluble EPS content in supernatant was determined using the standard curve. The experiment is independently repeated three times with three replicates in each group, with the negative control group using BHI bacterial suspension medium without drugs and the experimental group using BHI bacterial suspension medium containing Naringin.

#### 2.5.4 Effect of Naringin on acid production levels of three major cariogenic bacteria

##### 2.5.4.1 Effect of Naringin on acid production levels of mono-and multi-species cariogenic bacteria in planktonic state

Aliquots of overnight cultures of Monospecies (*S. mutans*, *S. sanguinis*, and *S. sobrinus*) and multispecies (1:1:1 ratio) were cultured with Naringin (8–0.125 mg/mL) in BHI with 1% sucrose. Within the 24 h cultivation period, at time points of 1, 2, 3, 4, 6, 8, 10, 12, 16, 20, and 24 h, equal amounts of the culture were centrifuged (8000 rpm, 4 °C, 30 min). The supernatant was collected, and the pH value was measured using a precision digital pH meter to plot the bacterial acid production curve (Mu et al., 2023). To compare the pH-lowering ability between groups, the pH difference before and after 24 h was calculated (ΔpH = initial pH − final pH). Lactate levels were determined using a commercial Lactate Assay Kit. Standards (0–0.5 mM) and samples (50 μL) were mixed with 50 μL WST-8 working solution in 96-well plates. After 30 min incubation at 37 °C in dark, absorbance was measured at 450 nm. Lactate concentrations were calculated from the standard curve. Culture pH was measured to calculate ΔpH (initial pH − final pH at 24 h).

##### 2.5.4.2 Effect of Naringin on acid production of mono-and multi-species cariogenic bacterial biofilms

Biofilms were formed on coverslips in 6-well plates with BHI containing 1% sucrose at 37 °C for 24 h. After washing with PBS, biofilms were treated with Naringin (MBIC-1/8MBIC) in fresh BHI (pH = 7.2) for 24 h. Supernatants were collected for lactate measurement (as in Section “2.5.4.1 Effect of Naringin on acid production levels of mono-and multi-species cariogenic bacteria in planktonic state”) and pH determination. All experiments were performed in triplicate with three independent replicates.

#### 2.5.5 Adhesion assay on hydroxyapatite (HAP) substrates

To evaluate the differential effects of Naringin on bacterial adhesion in mono-vs. multi-species biofilms, a fluorescence-based assay was performed on saliva-coated HAP substrates. HAP-coated plates were prepared using saliva-coated HAP. Bacteria were stained with BCECF/AM and co-incubated with Naringin (MIC-1/8MIC) on HAP plates. After washing, fluorescence intensity was measured at excitation/emission wavelengths of 495/525 nm. Adhesion inhibition rates were calculated as: [1 − (Fluorescence_drug_ − Fluorescence_background_) / (Fluorescence_control_ − Fluorescence_background_)] × 100%. Experiments were performed in triplicate and repeated independently three times. Adhesion patterns were visualized using fluorescence microscopy. This method allowed quantification of Naringin’s impact on bacterial attachment to HAP, a proxy for tooth enamel, in both mono-and multi-species contexts.

### 2.6 *In vivo* rat caries model with multi-species inoculation

#### 2.6.1 Animal model establishment and treatment

Forty-eight 15-days-old male SPF SD rats were randomly divided into 6 groups (8 rats/group) under specific pathogen-free conditions (25 ± 2 °C, 40%–60% humidity, 12 h light/dark cycle). The protocol was approved by the Institutional Animal Care and Use Committee (IACUC-JT-20240415-16) (Wu et al., 2024).

To eliminate endogenous microbiota, rats were fed water containing penicillin (200 μg/mL) and streptomycin (1500 μg/mL) with antibiotic-supplemented chow for 3 days. At day 18, rats were inoculated twice daily for 3 days with a mixed suspension of *S. mutans*, *S. sanguinis*, and *S. sobrinus* (1:1:1 ratio) onto molar surfaces. A cariogenic diet (Diet 2000) and 5% sucrose water were provided to all groups except the blank control from day 21 until sacrifice.

Drug treatment groups (500 μL, 2MIC; MIC and 1/2MIC of multi-species), caries model control group (CA, 500 μL, PBS) and positive control group (500 μL, CHX, 0.12%) and blank control group (vehicle-treated) received topical application on molar occlusal and buccolingual surfaces daily for 4 weeks using a micro-brush. Body weight and general conditions were monitored daily.

#### 2.6.2 Sample collection and analysis

##### 2.6.2.1 Oral microbiota analysis

At days 24, 30, 36, 42, and 48, dental plaque was collected with sterile swabs, diluted in PBS, and plated on Mitis Salivarius agar (with bacitracin 0.2 U/mL) for CFU counting and BHI agar for total microbiota.

##### 2.6.2.2 Caries lesion assessment

At day 49, jaws were harvested after CO_2_ euthanasia. Teeth were stained with 0.4% murexide for 12 h, sectioned mesiodistally, and scored via Keyes’ system (enamel only, slight dentin, moderate dentin, extensive dentin). Micro-CT (90 kV, 88 μA, 6 μm resolution) was used to reconstruct 3D molar structures.

##### 2.6.2.3 Biochemical and histopathological analysis

Serum samples were assayed for liver (ALT, AST, ALP) and kidney (BUN, CREA) function. Gingival and palatal tissues were fixed in 10% formalin, sectioned, and H&E-stained for histology.

### 2.7 Statistical analysis

All experimental data were presented as mean ± standard deviation (SD) from at least three independent replicates. Normality of data distribution was assessed using the Shapiro-Wilk test. For parametric data with homogeneous variances, one-way analysis of variance (ANOVA) was performed followed by Tukey’s *post-hoc* test. When variances were heterogeneous, Brown-Forsythe and Welch ANOVA were employed. Nonparametric data were analyzed using the Kruskal-Wallis test with Dunn’s *post hoc* multiple comparisons.

## 3 Results and discussion

### 3.1 Antimicrobial efficacy of Naringin against planktonic bacteria

#### 3.1.1 Mono-species growth inhibition

**FIGURE 1:**
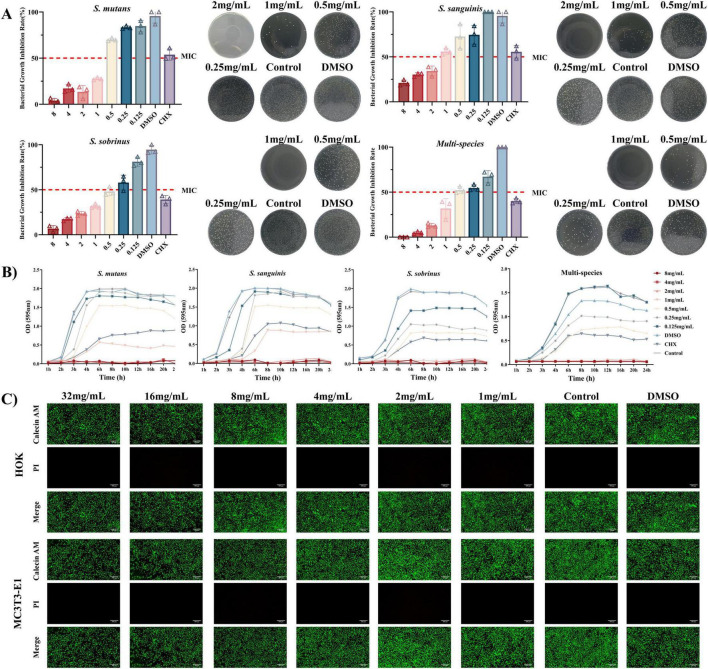
Antimicrobial efficacy and biosafety of Naringin against planktonic bacteria. (A) MIC and MBC of Naringin against mono-species and multi-species. (B) Time-kill curve. (C) Live/Dead cell staining. Scale bar: 200 μm.

Naringin exhibited potent concentration-dependent growth inhibition against *S. mutans*, *S. sanguinis*, and *S. sobrinus*, with MIC of 1.00, 1.00, and 0.50 mg/mL, respectively (Figure 1A). MBC were determined to be 2.00 mg/mL for *S. mutans*, 2.00 mg/mL for *S. sanguinis*, and 1.00 mg/mL for *S. sobrinus*, indicating complete killing at these concentrations. At concentrations ≥1 mg/mL, Naringin significantly reduced bacterial viability compared to the control and DMSO solvent control (*p* < 0.05). Colony-forming unit (CFU) counts mirrored these results, with high-concentration Naringin groups showing sparse colony formation comparable to CHX-treated samples.

Time-kill curve analysis revealed that Naringin at MIC significantly delayed the logarithmic phase and reduced bacterial proliferation in a time-dependent manner. For *S. mutans*, the logarithmic phase onset was delayed by 2 h, and the stationary-phase optical density (OD595) was reduced by 71.29% compared to the negative control group (Figure 1B). Specifically, the OD595 of *S. mutans* treated with 1.00 mg/mL Naringin reached a plateau of 0.570 ± 0.0071 at 6 h, whereas the untreated control reached 1.986 ± 0.0195 at 6 h. Similar trends were observed in *S. sanguinis* and *S. sobrinus*, where Naringin at 1.00 and 0.50 mg/mL, respectively, caused 3/2 h delay in the logarithmic phase and a 45%–60% reduction in stationary-phase OD. Additionally, the authors observed kinetic differences between Naringin and CHX via time-kill curves: CHX rapidly suppressed bacterial proliferation within 4 h, whereas Naringin-treated cultures exhibited delayed yet sustained growth inhibition. Taking *S. mutans* as an example, the optical density of the 2 mg/mL Naringin-treated group remained below 0.3 over 24 h, whereas the CHX-treated group showed a slight rebound in optical density after 12 h. This pattern was consistent across all tested bacterial species: Naringin not only exerted prolonged inhibition on bacterial growth but also prevented bacterial regrowth. Notably, Naringin exhibited species-specific antimicrobial efficacy: *S. sobrinus* was the most sensitive to it, while *S. sanguinis* showed the lowest sensitivity.

The concentration-dependent inhibition of Naringin may be attributed to its hydrophobic aglycone structure, which facilitates penetration into bacterial membranes and disruption of proton motive force. This finding is consistent with previous studies showing that flavonoids inhibit glucosyltransferase activity and reduce extracellular polysaccharide synthesis, thereby impairing bacterial adhesion and biofilm formation (Castellanos et al., 2024). The results highlight Naringin’s potential as a non-antibiotic agent for controlling cariogenic planktonic bacteria, particularly given its selective toxicity toward pathogens versus commensal oral microbiota. The time-dependent growth inhibition correlates with Naringin’s polyphenolic structure, which may interfere with bacterial membrane lipid bilayers and inhibit key metabolic enzymes, such as glucosyltransferases involved in exopolysaccharide synthesis (Gutiérrez-Venegas et al., 2019).

The concentration-dependent efficacy of Naringin underscores its therapeutic potential. While CHX requires lower concentrations for immediate effect, Naringin’s high-dose efficacy (2 mg/mL) combined with its low cytotoxicity positions it as a safer alternative for prolonged use. This advantage is particularly relevant in oral care, where CHX’s side effects (e.g., enamel staining, mucosal irritation) limit long-term application. Notably, Naringin’s ability to suppress biofilm precursors (e.g., *S. mutans* exopolysaccharides) further supports its utility in preventing dental caries, aligning with its dual mechanism of action: inhibiting both bacterial growth and biofilm formation. Furthermore, the low cytotoxicity of Naringin on human oral keratinocytes (HOK) and mouse pre-osteoblasts (MC3T3-E1) was confirmed at the tested antimicrobial concentrations (Figure 1C), supporting its favorable biosafety profile for potential oral applications.

#### 3.1.2 Multi-species consortium sensitivity

Naringin exhibited distinct antimicrobial susceptibility in the *S. mutans*/*S. sanguinis*/*S. sobrinus* consortium, with an MIC of 0.50 mg/mL and an MBC of 1.00 mg/mL. Contrary to the assumption of enhanced resistance in multi-species communities, comparative analysis with single-species profiles revealed a nuanced pattern: *S. mutans* and *S. sanguinis* each had an MIC of 1 mg/mL and MBC of 2 mg/mL, *S. sobrinus* showed an MIC of 0.5 mg/mL and MBC of 1 mg/mL, and the consortium’s MIC was equivalent to *S. sobrinus* (0.5 mg/mL) while lower than *S. mutans* and *S. sanguinis* (1 mg/mL). Its MBC (1 mg/mL) matched *S. sobrinus* and was lower than *S. mutans* and *S. sanguinis* (2 mg/mL), indicating that interspecies interactions do not uniformly enhance resistance but rather reshape the antimicrobial susceptibility landscape in a species-specific manner.

Time-kill curve analysis corroborated concentration-dependent inhibition of multi-species planktonic growth by Naringin: at 24 h, 0.50 mg/mL Naringin achieved 50% growth inhibition, comparable to the positive control CHX, whereas 0.125 mg/mL Naringin had negligible effects, similar to the DMSO solvent control (Figure 1B). Time-kill kinetics curve further demonstrated that the antimicrobial effect of Naringin against the consortium was time-dependent. At concentrations ≥0.5 mg/mL, Naringin significantly suppressed bacterial growth within 3 h, and this inhibitory effect persisted for 24 h.

The antimicrobial activity of Naringin against the consortium differed from that observed in single-species cultures, with the consortium exhibiting an MIC of 0.50 mg/mL – lower than the MIC of 1 mg/mL for *S. sanguinis* and *S. mutans* individually, and equivalent to the 0.5 mg/mL MIC of *S. sobrinus*. This pattern indicates that interspecies interactions among the three streptococci do not result in synergistic resistance enhancement, contrary to the common phenomenon where bacterial consortia often exhibit elevated antimicrobial tolerance via metabolic cross-feeding or extracellular matrix (ECM) protection (Xu et al., 2025). Notably, in the current study, the presence of *S. sanguinis*-an early colonizer known to modulate extracellular matrix composition – did not significantly impair Naringin’s efficacy within the multi-species consortium. This suggests that Naringin retains anti-biofilm activity even in a more ecologically relevant microbial context. Our findings further highlight that the outcome of interspecies interactions on antimicrobial efficacy is not uniform; it may enhance or diminish drug susceptibility depending on the specific species assemblage. The ability of Naringin to suppress the cariogenic consortium without resorting to broad-spectrum microbial elimination positions it as a promising candidate for ecological caries management, potentially via targeting inter-bacterial communication or EPS matrix formation, supports its potential application in preventing dental caries (Husain et al., 2021).

### 3.2 Naringin-mediated control of mono-species biofilms

#### 3.2.1 Biofilm formation inhibition (MBIC)

**FIGURE 2:**
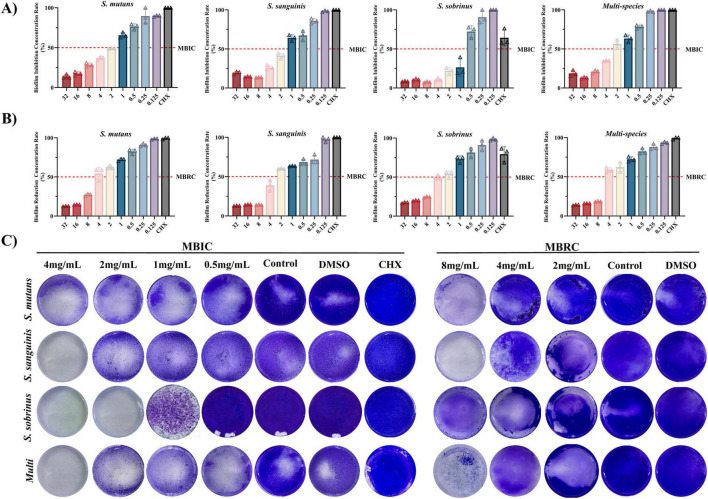
Efficacy of Naringin against cariogenic biofilms. (A) MBIC of Naringin against mono-species and multi-species. (B) MBRC of Naringin against mono-species and multi-species. (C) Crystal violet staining.

Naringin demonstrated potent inhibition of early-stage biofilm formation, with MBIC values of 2.00 mg/mL for *S. mutans*, 2.00 mg/mL for *S. sanguinis*, and 1.00 mg/mL for *S. sobrinus* (Figure 2A). The dose-response relationship revealed that Naringin suppressed biofilm development in a concentration-dependent manner: at 4 mg/mL, biofilm biomass was reduced by >70% for all three species compared to the DMSO control, while 0.5 mg/mL Naringin exhibited minimal inhibitory effects (<20%). Notably, *S. sobrinus* displayed the highest sensitivity to Naringin, with complete inhibition of biofilm initiation at 1 mg/mL. The MBIC values correlated with planktonic MIC data, suggesting that Naringin inhibits biofilm formation primarily by suppressing bacterial growth.

#### 3.2.2 Pre-formed biofilm disruption (MBRC)

Against mature biofilms, Naringin exhibited consistent minimum biofilm reduction concentrations (MBRC) of 4.00 mg/mL for all three single species: *S. mutans*, *S. sanguinis*, and *S. sobrinus* (Figure 2B). The dose-dependent disruption efficacy showed that 8 mg/mL Naringin achieved >75% reduction in biofilm biomass for each species, whereas concentrations below 1 mg/mL had negligible effects (<15%). Notably, at the high concentration of 8 mg/mL, *S. sanguinis* mature biofilms maintained relatively higher susceptibility to Naringin compared to *S. mutans* and *S. sobrinus*, with a more pronounced reduction in biofilm biomass. This species-specific response to elevated Naringin concentrations contrasts with the uniform MBRC (4.00 mg/mL) across all three species. The dose-dependent disruption of mature biofilms was visually confirmed by crystal violet staining (Figure 2C).

The higher MBRC values relative to MBIC indicate that mature biofilms-characterized by dense ECM and recalcitrant bacterial communities-require greater concentrations of Naringin for effective disruption. This phenomenon is consistent with the protective role of the biofilm matrix, which limits antimicrobial penetration and shields embedded bacteria from environmental stressors. For *S. mutans* and *S. sobrinus*, the dense network of EPS synthesized via glucosyltransferases (GTFs) likely contributes to their higher MBRC, as EPS forms a structural scaffold that strengthens biofilm integrity. Naringin’s ability to reduce EPS production in planktonic cells may indirectly weaken this matrix, facilitating biofilm dispersion at higher concentrations. Mechanistically, Naringin-induced disruption of mature biofilms may involve dual targeting of bacterial cells and the ECM.

### 3.3 Mechanistic insights into biofilm disruption

#### 3.3.1 Tri-species biofilm architecture and biomass

**FIGURE 3:**
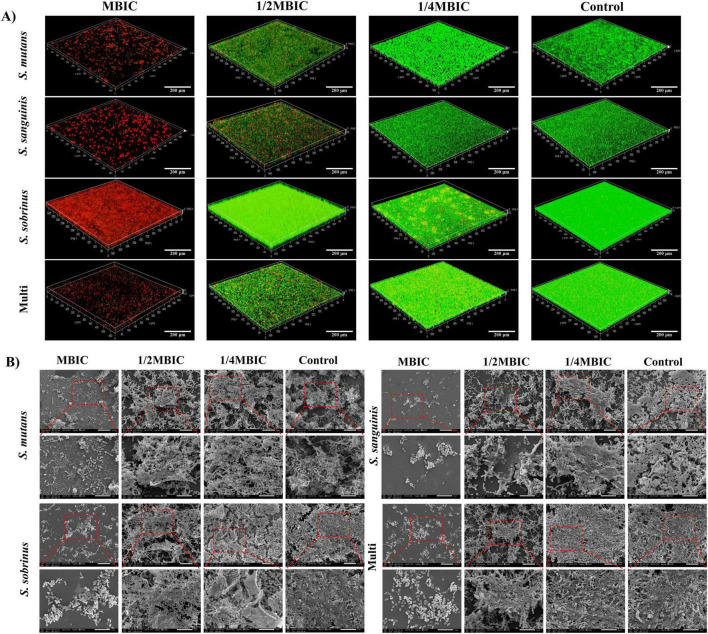
Tri-species biofilm architecture and biomass. (A) Live/dead bacteria staining. Scale bar: 200 μm. (B) Ultrastructural alterations by SEM. Scale bar: 10 μm.

The tri-species biofilm model exhibited a dense, hierarchical structure with distinct microcolony organization, as confirmed by confocal laser scanning microscopy (CLSM; Figure 3A). Co-cultures of *S. mutans*/*S. sanguinis*/*S. sobrinus* formed a complex matrix with interwoven bacterial aggregates, where *S. sanguinis* occupied the basal layer (adherent to the substratum), *S. mutans* formed intermediate microcolonies, and *S. sobrinus* localized in the upper ECM (Liu et al., 2022). Naringin treatment disrupted this architecture in a concentration-dependent manner. At 1 mg/mL (1/2 × MBIC of *S. mutans*), the biofilm biomass was reduced by 42% (*P* < 0.05), with CLSM showing fragmented microcolonies and reduced ECM continuity. At 2 mg/mL (1 × MBIC of multi-species), tri-species biomass reduction exceeded 70% (*P* < 0.001), accompanied by loss of stratification-*S. sanguinis* basal layers disassembled, and *S. mutans*/*S. sobrinus* aggregates dispersed into smaller clusters. Notably, the tri-species biofilm required higher Naringin concentrations to achieve comparable disruption to mono-species biofilms (e.g., *S. sobrinus* MBIC = 1 mg/mL vs. tri-species MBIC = 2 mg/L), highlighting enhanced resistance in mixed communities.

#### 3.3.2 Ultrastructural alterations by SEM

Scanning electron microscopy revealed distinct ultrastructural changes in Naringin-treated mono-and multi-species biofilms, providing mechanistic insights into biofilm disruption. For mono-species biofilms, *S. mutans* exhibited the most pronounced morphological damage: at 1/2MBIC, the dense, cohesive biofilm matrix appeared fragmented, with bacterial chains dissociating into individual cells (Figure 3B). Higher concentrations induced severe membrane wrinkling and cytoplasmic leakage, consistent with loss of cell integrity. Similarly, *S. sobrinus* biofilms treated with MBIC Naringin showed reduced microcolony density, with scattered bacteria lacking the characteristic basal layer adhesion observed in untreated controls.

In multi-species biofilms (*S. mutans*/*S. sanguinis*/*S. sobrinus*), Naringin-induced damage was hierarchical and concentration-dependent. At 1/2MBIC, the stratified architecture-characterized by *S. sanguinis* basal clusters, *S. mutans* intermediate layers, and *S. sobrinus* ECM-embedded aggregates-was disrupted: the basal layer detached from the substratum, and interspecies bridges (likely composed of EPS) were fractured. At MBIC, extensive matrix degradation exposed individual bacteria, many of which exhibited membrane blebbing and irregular morphologies, indicating widespread cellular stress. Multi-species biofilms required higher Naringin concentrations than mono-species biofilms to achieve equivalent ultrastructural damage.

#### 3.3.3 Inhibition of acid production

**FIGURE 4:**
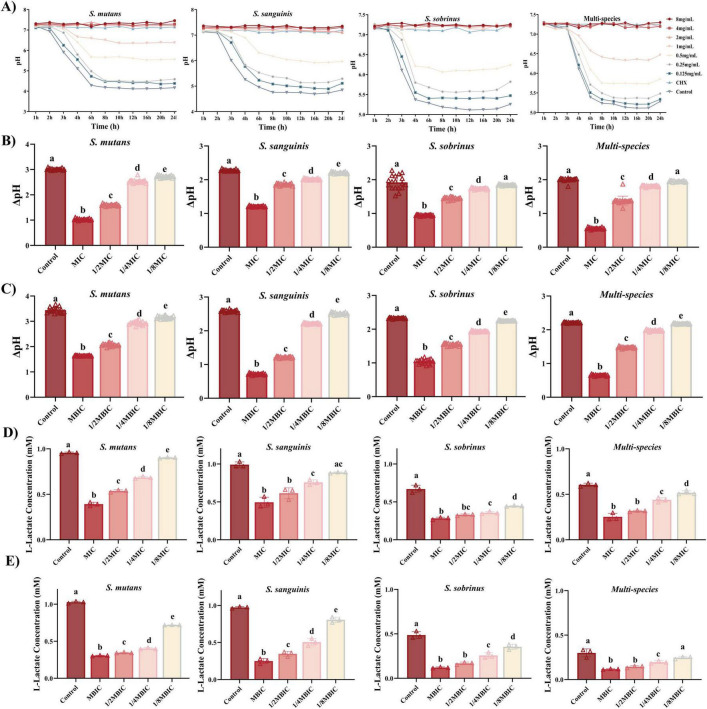
Naringin inhibits acid production of cariogenic bacteria. (A) Acid production curves of planktonic bacteria over 24 h under Naringin treatment. (B) Acid production levels (ΔpH) of planktonic bacteria after Naringin treatment. (C) Acid production levels (ΔpH) of bacterial biofilms after Naringin treatment. (D) Lactate production in planktonic bacterial cultures following Naringin exposure. (E) Lactate production in cariogenic biofilms after Naringin treatment. Dissimilar letters indicate significantly different values (*P* < 0.05).

Acid production is a pivotal virulence factor of cariogenic biofilms, as sustained acidification drives enamel demineralization. Naringin exhibited concentration – dependent inhibition of acid production in both planktonic and biofilm states, with distinct efficacy between mono-species and multi-species communities. Time-course pH monitoring (Figure 4A) revealed that Naringin delayed and attenuated acidification across all tested strains. For *S. mutans*, the untreated control exhibited a rapid pH drop from 7.2 to 4.3 within 6 h, while 0.5 mg/mL Naringin maintained pH above 5.5 for 24 h (consistent with the critical threshold for enamel demineralization). A similar trend was observed for *S. sanguinis*, where 0.5 mg/mL Naringin reduced the ΔpH drop by 32% compared to the control (2.2–1.5). Notably, the multi-species consortium displayed enhanced acidogenic resilience: even 1 mg/mL Naringin failed to fully prevent pH from falling below 6.5 by 24 h, though the rate of acidification was halved relative to the untreated group. This aligns with previous observations that interspecies metabolic cross-feeding in mixed biofilms enhances acid production persistence.

In planktonic cultures (Figure 4B), Naringin suppressed acid production in a dose-dependent manner, with MIC concentrations inducing the most pronounced effects. *S. mutans* showed a 67% reduction in ΔpH at MIC (1 mg/mL) compared to the control (*P* < 0.001), while *S. sobrinus* exhibited a 51% reduction at its MIC (0.5 mg/mL). The inhibitory efficacy correlated with growth suppression, suggesting Naringin may disrupt glycolytic pathways-consistent with reduced lactate dehydrogenase (LDH) activity observed in flavonoid-treated streptococci (Xu et al., 2011). Importantly, 1/2MIC concentrations still significantly inhibited acid production (*P* < 0.05), indicating a specific targeting of metabolic activity beyond mere growth inhibition. Biofilms required higher Naringin concentrations to achieve comparable acid inhibition, reflecting their intrinsic resistance (Figure 4C).

Quantitative analysis of lactate production provided direct evidence for Naringin’s anti-acidogenic properties. In planktonic cultures (Figure 4D), Naringin dose-dependently reduced lactate accumulation across all strains. For *S. mutans*, lactate production at MIC (1 mg/mL) was decreased by 59.0% compared to the untreated control (0.392 vs. 0.956 mM, *P* < 0.001). Similarly, *S. sobrinus* showed a 57.7% reduction at its MIC (0.5 mg/mL), with lactate levels dropping from 0.670 to 0.283 mM. The multi-species consortium demonstrated intermediate susceptibility, with MIC treatment achieving a 58.3% lactate reduction (0.252 vs. 0.605 mM). Notably, even sub-MIC concentrations (1/2–1/8MIC) significantly suppressed lactate production (*P* < 0.05), confirming Naringin’s potent interference with bacterial acid metabolism.

Quantitative analysis of lactate production in mature biofilms confirmed that Naringin concentration-dependently suppressed this key cariogenic virulence trait across all tested strains (Figure 4E), though with distinct efficacies. *S. mutans* exhibited the highest baseline acidogenicity (1.027 mM) and was strongly inhibited at MIC (0.307 mM, 70.1% reduction), while *S. sobrinus*, with the lowest baseline (0.486 mM), was the most susceptible, showing a 75.1% reduction (0.121 mM) at MIC. The primary pathogen *S. sanguinis* was also effectively suppressed at MIC (0.249 mM vs. control 0.974 mM). The multi-species consortium demonstrated unique resilience; despite a low baseline (0.300 mM), its lactate levels remained notably stable across sub-MIC treatments (0.247–0.308 mM), underscoring the enhanced tolerance afforded by inter-species interactions within the biofilm community.

The significant suppression of lactate production, observed even at sub-MIC concentrations where bacterial growth is not fully arrested, strongly suggests that Naringin directly targets the acid-generating metabolic pathway in addition to its growth-inhibitory effect. This anti-acidogenic activity is particularly evident in the dose-dependent reduction of lactate accumulation across both planktonic and biofilm states. We hypothesize that Naringin may interfere with the activity of lactate dehydrogenase (LDH) – the key enzyme responsible for pyruvate-to-lactate conversion in the glycolytic pathway-or potentially regulate the expression of its encoding gene (LDH). This mechanistic inference aligns with previous reports on flavonoid-mediated inhibition of streptococcal acidogenicity (Xu et al., 2011), and provides a plausible molecular explanation for the observed delay in pH drop and reduction in final lactate yield. While direct measurement of LDH activity and LDH expression will be essential to validate this hypothesis, the consistent anti-acidogenic phenotype across multiple strains strengthens the proposition that Naringin disrupts the core glycolytic metabolism of cariogenic bacteria.

These findings collectively demonstrate that Naringin effectively suppresses the cariogenic virulence trait of acid production. The concentration-dependent reduction in lactate accumulation, particularly at sub-inhibitory concentrations, indicates a direct anti-acidogenic effect that complements its growth-inhibitory activity. Although multi-species biofilms exhibited notable tolerance, the significant suppression of acid production observed under both planktonic and biofilm conditions supports the further investigation of Naringin as a promising anti-caries agent.

#### 3.3.4 Inhibition of EPS production

**FIGURE 5:**
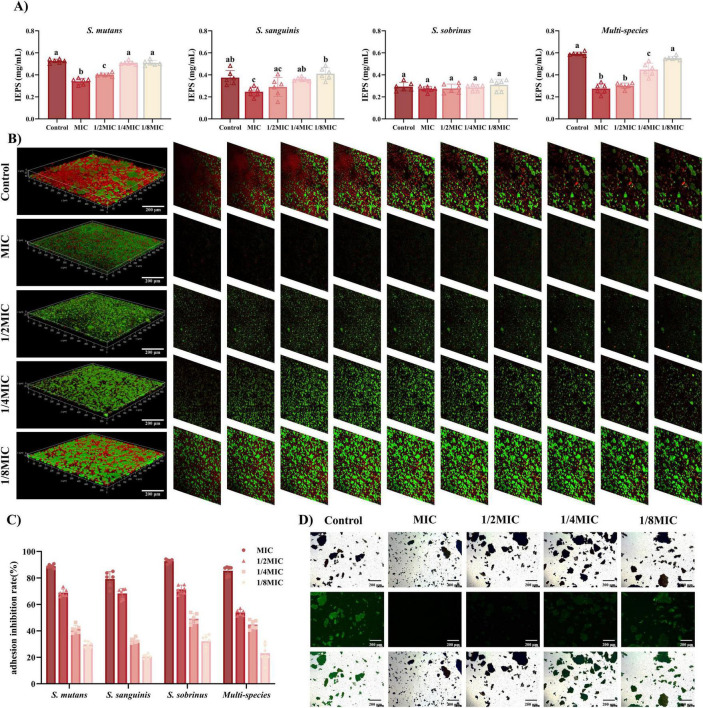
Naringin inhibits the virulence of cariogenic bacteria. (A) Effect of Naringin on water-insoluble extracellular polysaccharides (EPS) synthesis in planktonic bacteria. (B) CLSM analysis of EPS (red) and bacterial cells (green) in biofilms treated with Naringin. Scale bar: 200 μm. (C) Naringin intervention significantly inhibited bacterial adhesion to saliva-coated HAP in a dose-dependent manner, with the MIC group showing the highest inhibition rate. (D) Fluorescence microscopy images showing bacterial adhesion to saliva-coated HAP surfaces. Dissimilar letters indicate significantly different values (*P* < 0.05). Scale bar: 200 μm.

Extracellular polysaccharides (EPS), primarily composed of α-1,3-linked glucans, are critical for maintaining the structural integrity of cariogenic biofilms and enhancing acid retention. Naringin exhibited species-specific inhibition of EPS production in planktonic cultures, with distinct efficacy across streptococcal species (Figure 5A). For *S. mutans*, Naringin suppressed EPS synthesis in a dose-dependent manner: MIC (1 mg/mL) reduced EPS production by 35% compared to the untreated control, while 1/2MIC still achieved a 24% reduction. In contrast, *S. sanguinis* showed significant inhibition only at MIC, with EPS levels decreasing by 34%. Notably, Naringin had no significant effect on EPS production in *S. sobrinus* (1 mg/mL) across all tested concentrations (*P* > 0.05), indicating a species-specific mechanism.

The selective inhibition of EPS in streptococci strongly suggests that Naringin interferes with GTF activity-a key enzyme complex responsible for glucan synthesis, particularly GtfB and GtfC in *S. mutans* (Pandit et al., 2024), which are essential for EPS-mediated biofilm cohesion. While the present study did not directly measure *gtf* gene expression, the observed dose-dependent reduction in insoluble EPS and the concomitant disruption of biofilm integrity (Section “3.3.2 Ultrastructural alterations by SEM”) are consistent with the inhibition of GTF function at the enzymatic level. Mechanistically, the decrease in EPS weakens the extracellular matrix, thereby reducing bacterial co-aggregation and diminishing the “matrix shield” that mitigates acid trapping and antimicrobial penetration. This provides a plausible explanation for the synergistic effects of Naringin on both biofilm biomass and acidogenicity. In contrast to broad-spectrum agents such as Turkish gall extract (TGE) (Xu et al., 2023), which non-specifically targets bacterial membranes through polyphenolic action, Naringin appears to exert a more targeted effect on streptococcal GTF-mediated EPS assembly, which may better preserve commensal flora-a vital consideration for maintaining ecological balance in the oral microbiome.

#### 3.3.5 Glycocalyx disruption via fluorescent staining in multi-species biofilms

Fluorescent staining of EPS combined with 3D CLSM visualization revealed concentration-dependent disruption of the glycocalyx in the *S. mutans*/*S. sanguinis*/*S. sobrinus* consortium, with distinct structural alterations across Naringin concentrations (Figure 5B). This visualization provided direct evidence of Naringin’s ability to disrupt the EPS matrix.

In untreated multi-species biofilms, CLSM images showed a dense, interconnected EPS matrix with stratified organization: *S. sanguinis* embedded in the basal EPS layer, *S. mutans* microcolonies surrounded by a thick EPS sheath, and *S. sobrinus* distributed in the upper EPS network. At MBIC (2 mg/mL), the EPS matrix exhibited extensive fragmentation: fluorescence intensity decreased by 53% compared to controls, with loss of stratification and detachment of *S. mutans* clusters from the substratum. The 3D reconstruction revealed discontinuous EPS patches, particularly in the intermediate layer occupied by *S. mutans*. At 1/2MBIC (1 mg/mL), partial preservation of the basal EPS layer was observed, but the upper EPS network (predominantly *S. sobrinus*-associated) showed 38% reduced fluorescence, with *S. mutans* microcolonies appearing less cohesive. The 1/4MBIC (0.5 mg/mL) group displayed milder disruption: EPS thickness decreased by 19% (*P* < 0.05), but structural integrity was largely maintained, with only scattered gaps in the matrix.

The collapse of the EPS scaffold not only compromises biofilm mechanical stability but also disrupts the metabolic symbiosis between streptococcal species, which is partially mediated by spatial co-localization within the glycocalyx. Furthermore, the persistence of a basal EPS layer at sub-MBIC levels indicates a potential threshold for complete matrix disintegration, highlighting how partial EPS reduction may suffice to impair biofilm function without fully eliminating the matrix. This structural vulnerability, induced by Naringin, likely exacerbates bacterial sensitivity to environmental stresses and enhances the penetration of antimicrobials, providing a mechanistic link between matrix disruption and the observed reductions in biofilm viability and acidogenesis.

#### 3.3.6 Bacterial adhesion inhibition on HAP

Naringin demonstrated concentration-dependent inhibition of both mono-species and multi-species bacterial adhesion to HAP, with distinct species-specific and community-level dynamics (Figure 5C). The multi-species consortium (*S. mutans*/*S. sanguinis*/*S. sobrinus*) showed robust adhesion to saliva-coated HAP in untreated controls, with an adhesion rate of 82.6%. Naringin treatment significantly reduced this rate in a dose-dependent manner: at MIC_*multi–species*_ (1 mg/mL), adhesion was inhibited by 68.3%; at 1/2MIC, inhibition dropped to 41.5%; and at 1/4MIC, only 19.7% inhibition was observed. In contrast to multi-species biofilms, mono-species adhesion to HAP was more susceptible to Naringin: *S. mutans* adhesion was inhibited by 78.5% at MIC, compared to 68.3% in the consortium. This difference highlights the role of interspecies synergies in enhancing adhesion resilience *S. sanguinis* facilitates co-aggregation by providing binding sites for *S. mutans* and *S. sobrinus*, a process partially blocked by Naringin (Kolenbrander et al., 2006). Fluorescence microscopy of BCECF/AM-stained multi-species biofilms revealed striking morphological changes after Naringin treatment (Figure 5D). Untreated controls formed dense, continuous bacterial clusters across the HAP surface, with *S. mutans* and *S. sobrinus* embedded in an EPS matrix (green fluorescence co-localized with EPS staining). At MIC, bacterial aggregates fragmented into discrete microcolonies, with a 63% reduction in surface coverage. At 1/2MIC, sparse adhesion was observed, primarily as single cells or small chains, indicating impaired interspecies co-aggregation. Notably, *S. sanguinis*-which mediates initial adhesion via salivary protein receptors-was nearly absent from HAP surfaces at MIC, suggesting Naringin targets its adhesion machinery preferentially.

### 3.4 *In vivo* efficacy and safety in rat caries model

#### 3.4.1 Phenotypic and quantitative assessment of carious lesions

**FIGURE 6:**
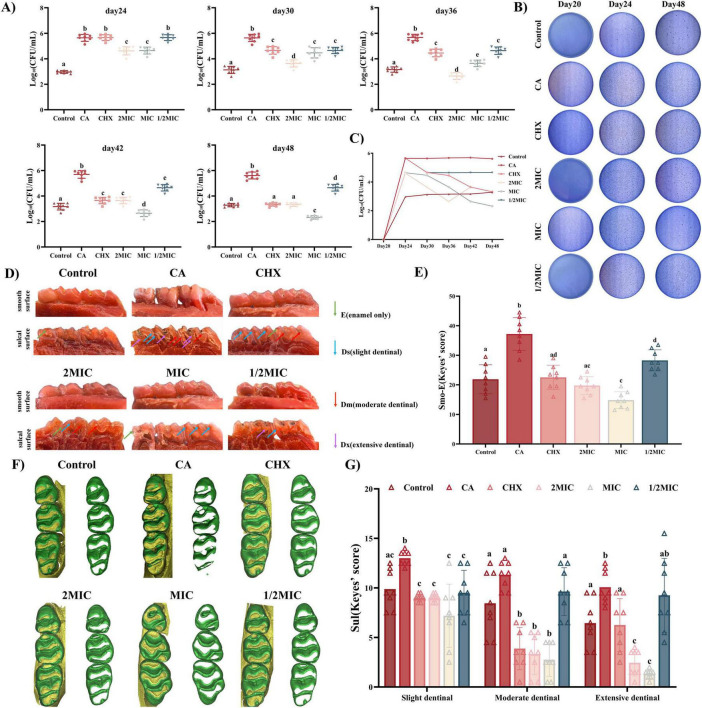
Evaluation of the efficacy of Naringin in the prevention and treatment of dental caries in rats. (A) Statistical analysis of surviving bacterial colonies after Naringin. (B) Representative images of surviving bacterial colonies on MSB agar plates for different treatment groups at 20, 24 and 48 days. (C) CFU counting for surviving bacterial colonies at 17, 24, 30, 36, 42, and 48 days. (D) Representative images of carious lesions stained by murexide on the smooth surface and sulcal surface with teeth of different severities. (E,G) Statistical analysis of Keyes’ scoring on the smooth surface and sulcal surface. (F) 3D reconstruction of micro-CT images of maxillary molars in different groups. Naringin treatment groups (2MIC, MIC, and 1/2MIC), Caries model control group (CA, cariogenic challenge + vehicle), Positive control group (CHX, cariogenic challenge + 0.12% chlorhexidine), and Blank control group (Control, no challenge, no treatment). Dissimilar letters indicate significantly different values (*P* < 0.05).

Naringin significantly reduced caries progression in rats, with concentration-dependent efficacy and a striking optimal effect at MIC, as evidenced by phenotypic observation, Keyes scoring, and Micro-CT analysis (Figure 6).

The inhibitory effect of Naringin on dental caries was systematically evaluated using Keyes’ scoring system, which classifies carious lesions into four grades based on depth: enamel-only (E), slight dentinal (Ds, ≤1/4 dentin), moderate dentinal (Dm, 1/4–3/4 dentin), and extensive dentinal (Dx, >3/4 dentin). Gross observation of rat dentition (Figure 6D) revealed that the caries model control group (CA, cariogenic challenge + PBS) exhibited severe dental caries, with extensive enamel discoloration, cavitation on smooth surfaces and sulcal surface. In contrast, Naringin-treated groups showed dose-dependent improvement, with the MIC group displaying the most favorable outcomes: smooth surfaces and sulcal surface remained largely intact-comparable to the CHX group, the gold standard anti-caries agent. Quantitative Keyes scoring confirmed these observations (Figures 6E, G). For smooth surface caries, the caries model control group had a mean Keyes score of 37.19 ± 1.96, while the MIC group reduced this to 14.8 ± 1.004, significantly lower than both the 2MIC group (19.75 ± 1.065) and the 1/2MIC group (28.75 ± 1.244). For sulcal surface caries, categorized into Ds, Dm, Dx, the caries model control group consistently exhibited the highest caries scores across all three categories, indicating severe lesion progression. In contrast, the MIC group showed markedly lower scores than the Model, 2MIC, and 1/2MIC groups in every category, demonstrating superior caries-inhibiting efficacy. The CHX group displayed comparable performance to the MIC group but with a trend toward greater inter-individual variability. Both the 2MIC and 1/2MIC groups had higher caries scores than the MIC group: the 2MIC group likely reflected over-inhibition-induced microbial imbalance (as discussed previously), while the 1/2MIC group was constrained by inadequate biofilm penetration, consistent with earlier observations of its diminished biofilm-disrupting ability. The control group maintained the lowest scores, serving as the baseline for healthy sulcal surfaces. 3D Micro-CT reconstruction (Figure 6F) provided quantitative evidence of enamel preservation.

The superior efficacy of the MIC group aligns with its balanced modulation of oral microbiota and targeted inhibition of cariogenic virulence factors. At MIC, Naringin effectively suppressed *S. mutans*/*S. sanguinis*/*S. sobrinus* growth (Section “3.1.2 Multi-species consortium sensitivity”), disrupted biofilm architecture (Section “3.3.2 Ultrastructural alterations by SEM”), reduced acid production (Section “3.3.3 Inhibition of acid production”), and inhibited adhesion to enamel – like HAP (Section “3.4 *In vivo* efficacy and safety in rat caries model”) – collectively blocking the “adhesion-biofilm-acid” cascade driving caries. This multi-targeted action was corroborated by Keyes scoring, where reduced lesion severity correlated with lower plaque biomass in rat saliva. The 2MIC group’s suboptimal performance likely stems from excessive microbiota disruption: high-dose Naringin not only inhibited streptococci but also depleted commensal bacteria (e.g., *S. sanguinis*), allowing opportunistic fungi (*C. albicans*) to dominate-consistent with previous reports (Li Q. et al., 2023) that broad-spectrum antimicrobials can induce fungal overgrowth in oral niches. Fungal-bacterial synergies may have exacerbated enamel demineralization, offsetting Naringin’s direct anti-caries effects.

Conversely, the 1/2MIC group’s limited efficacy reflects insufficient inhibition of biofilm maturation: despite partial reduction in initial adhesion (Section “3.4 *In vivo* efficacy and safety in rat caries model”), residual bacteria formed acidogenic biofilms, as evidenced by persistent low pH in plaque. This aligns with *in vitro* data showing 1/2MIC Naringin fails to suppress EPS production (Section “3.3.4 Inhibition of EPS production”) or acidogenesis (Section “3.3.3 Inhibition of acid production”) in multi-species biofilms. In summary, Naringin mitigates rat caries via multi-targeted inhibition of cariogenic processes, with MIC representing the optimal concentration.

#### 3.4.2 Oral microbiota and safety profiles

From Day 15 to Day 48, the Caries model control group (CA) displayed exponential plaque growth (Figures 6A, B), peaking at Day 24 (log_10_ CFU/mL = 5.5685) and subsequently stabilizing, remaining hyperabundant (log_10_ CFU/mL ≥ 5.0). In contrast, the Control group maintained a stable low burden (log_10_ CFU/mL = 2.9618). Naringin-treated groups showed distinct time-concentration profiles: MIC group: Plaque load continuously decreased post-Day 24, reaching 2.319 log_10_ CFU/mL at Day 48-significantly lower than CHX (3.3211, *P* < 0.05) and 2MIC (3.3199, *P* < 0.05) groups.

This superior efficacy of the MIC group is consistent with the differential antimicrobial activity observed for Naringin *in vitro*: *S. sobrinus*, being more sensitive to Naringin than *S. mutans* and *S. sanguinis*, was effectively suppressed alongside *S. mutans* (the primary drivers of high plaque load in the caries model control group), while the commensal *S. sanguinis* – which exhibits higher tolerance to Naringin than cariogens demonstrated higher tolerance. This functional profile, inferred from plaque biomass dynamics, is indicative of a selective modulation that may help maintain a balance in the oral microbiota, avoiding the complete microbial collapse observed in the 2MIC group. Conversely, 2MIC concentrations induced over-inhibition, indiscriminately suppressing both cariogens and commensals, thereby creating ecological niches for opportunistic pathogens. This likely contributed to the late-stage plaque rebound in the 2MIC group, undermining its long-term efficacy compared to the MIC group.

The superior caries reduction observed in the MIC group directly correlates with its ability to stably modulate the microbiota. Specifically, Naringin at MIC exerted sustained suppression of plaque during the critical caries progression window (Day 24–36), when lesions in the caries model control group accelerated; this aligns with its capacity to reduce acid production (Section “3.3.3 Inhibition of acid production”) and enhance enamel preservation (Section 3.4.1). In contrast, while CHX transiently reduced plaque (Day 24–30), its broad-spectrum activity induced greater microbiota fluctuations-evidenced by higher inter-individual variance in plaque counts (*P* < 0.05 vs. MIC) – increasing the risk of relapse. Thus, Naringin’s selective modulation at MIC provides a more sustainable microenvironment for enamel protection.

Collectively, these data highlight Naringin’s unique capacity to-reset the oral microbiota toward a cariostasis-promoting state at MIC, avoiding the pitfalls of over- or under-inhibition. This concentration-dependent microbiota modulation underpins its *in vivo* anti-caries efficacy, reinforcing MIC as the therapeutically optimal dose.

#### 3.4.3 Histopathological analysis of organs and mucosa

**FIGURE 7:**
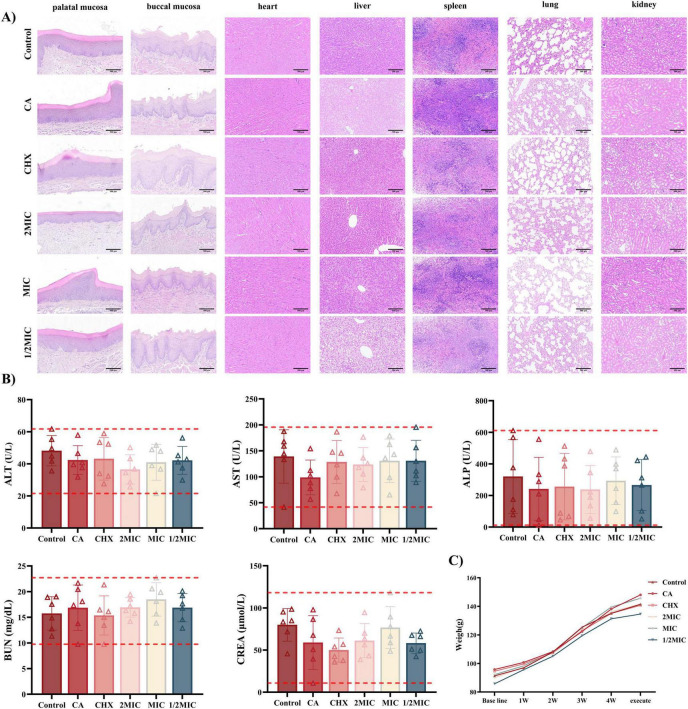
Biocompatibility of Naringin *in vivo*. (A) H&E staining of the oral mucosa and major organs from different groups. Scale bar: 500 μm. (B) Blood biochemistry analysis of liver function and kidney function. (C) Body weight changes of rats with different treatments. Groups: Naringin treatment groups (2MIC, MIC, and 1/2MIC), Caries model control group (CA, cariogenic challenge + vehicle), Positive control group (CHX, cariogenic challenge + 0.12% chlorhexidine), and Blank control group (Control, no challenge, no treatment).

Hematoxylin and eosin (HE) staining of oral mucosa (buccal and palatal) and major organs (heart, liver, spleen, lung, and kidney) revealed intact tissue structures across all groups, with Naringin-treated rats showing no evidence of toxicity or irritation (Figure 7A). The Control group displayed intact oral mucosa characterized by well-stratified epithelium, regular rete ridge formation, and absence of submucosal inflammation. Naringin-treated groups (2MIC, MIC, 1/2MIC) exhibited comparable architecture: palatal mucosa maintained a smooth epithelial surface, and buccal mucosa showed normal cellular organization, with no signs of epithelial damage or inflammatory cell infiltration.

About visceral Organs, all groups showed normal alignment of myocardial fibers, intact intercalated disks, and no necrosis or inflammation, confirming preserved cardiac structure. The Control and Naringin-treated groups maintained intact hepatic lobules with polygonal hepatocytes, clear sinusoidal spaces, and no fatty infiltration. However, the CHX group exhibited sporadic lymphocytic infiltration in portal areas. Normal white pulp and red pulp were observed in all groups, with no abnormal lymphoid hyperplasia or depletion, suggesting preserved immune homeostasis in Naringin-treated rats. Alveolar structures remained intact in Naringin-treated groups, with no interstitial fibrosis or inflammatory cell aggregation. Glomeruli and renal tubules exhibited normal morphology across all groups, with no hyaline casts (indicative of proteinuria) or tubular necrosis, consistent with stable renal function (Figure 7B). The caries model control group (caries-induced) showed no organ-specific pathology, confirming that cariogenic challenge alone did not induce systemic tissue damage. Naringin-particularly at MIC-maintained tissue homeostasis in both mucosa and organs, whereas CHX induced subtle but detectable mucosal irritation (buccal edema) and hepatic inflammation, reinforcing Naringin’s superior safety profile for long-term oral application.

Serum biochemical analysis (Figure 7B) demonstrated that liver function markers – alanine transaminase (ALT) and aspartate transaminase (AST) – stayed within normal ranges across all Naringin groups (2MIC, MIC, 1/2MIC). For renal function, serum urea nitrogen (BUN) and creatinine (Cr) levels remained stable in all Naringin groups, with no statistical difference from the Control group (*P* > 0.05). The caries model control group also showed normal renal indices despite cariogenic challenge, ruling out disease-related systemic toxicity.

Longitudinal weight monitoring (Figure 7C) revealed that Naringin-treated rats-maintained growth trajectories comparable to the Control group over 48 days. The MIC group gained weight steadily, similar to the Control group. In contrast, the CHX group exhibited slower growth, attributed to its well-documented side effects of taste alteration and reduced food intake (Bernabe et al., 2025). The caries model control group showed moderate weight gain, indicating that cariogenic challenge alone did not impair systemic health, while Naringin intervention preserved normal growth.

Collectively, these data demonstrate that Naringin-particularly at MIC-balances anti-caries efficacy with systemic and local safety. Its lack of organ toxicity, mucosal irritation, and adverse effects on growth contrasts with CHX, highlighting its potential as a safe alternative for long-term oral care. These findings align with previous reports on the biocompatibility of natural flavonoids, supporting their translational relevance in preventive dentistry.

## 4 Discussion

The species-specific antimicrobial profile of Naringin-demonstrating greater efficacy against the strong cartogens *S. mutans* and *S. sobrinus* than the commensal *S. sanguinis in vitro*-provided a mechanistic basis for the favorable oral microbiota outcomes observed *in vivo*. Specifically, topical application of Naringin at the MIC (1 mg/mL) in our rat caries model effectively suppressed plaque overgrowth and reduced carious lesions, outperforming both the 2MIC group and CHX in mitigating lesions while concurrently preserving microbial diversity. This functional profile, characterized by targeted virulence suppression without broad-spectrum eradication, aligns with the principles of ecological plaque control. While direct quantification of species abundance within the complex biofilm was beyond the scope of this study, the concordance between *in vitro* selectivity and *in vivo* microbiota stabilization strongly supports its ecological potential. Future research employing techniques such as metagenomic sequencing or FISH will be crucial to definitively confirm the compositional shifts. Beyond its ecological promise, Naringin’s natural origin and GRAS status offer regulatory advantages. The successful translation of these findings, however, hinges on overcoming challenges related to its short oral half-life through innovative formulation strategies, such as the development of mucoadhesive nanoparticles. Subsequent research priorities should include clinical trials in high-caries-risk cohorts and the exploration of synergistic combinations with remineralizing agents to achieve comprehensive caries management.

## Data Availability

The original contributions presented in this study are included in this article/supplementary material, further inquiries can be directed to the corresponding authors.
